# Does being physically active prevent future disability in older people? Attenuated effects when taking time-dependent confounders into account

**DOI:** 10.1186/s12877-017-0657-3

**Published:** 2017-12-21

**Authors:** Stefan H. Kreisel, Christian Blahak, Hansjörg Bäzner, Michael G. Hennerici

**Affiliations:** 1 Geriatric Psychiatry; Department of Psychiatry and Psychotherapy, Evangelisches Klinikum Bethel, Bielefeld, Germany; 2 Department of Neurology, Universitäts Medizin Mannheim, Mannheim, Germany; 3 Department of Neurology, Klinikum Stuttgart, Stuttgart, Germany

**Keywords:** Physical activity, Marginal structural models, Causal inference, Age-related white matter lesions, Disability, Longitudinal observational studies

## Abstract

**Background:**

Causal experimental evidence that physical activity prevents disability in older people is sparse. Being physically active has nonetheless been shown to be associated with disability-free survival in observational studies. Observational studies are, however, prone to bias introduced by time-dependent confounding. Time-dependent confounding occurs when an exposure (e.g. being physically active at some time-point) potentially affects the future status of a confounder (such as depression sometime later), and both variables have an effect on latter outcome (i.e. disability). “Conventional” analysis with e.g. Cox-regression is the mainstay when analyzing longitudinal observational studies. Unfortunately, it does not provide unbiased estimates in the presence of time-dependent confounding. Marginal structural models (MSM) – a relatively new class of causal models – have the potential to adequately account for time-dependent confounding.

Here we analyze the effect of older people being physically active on disability, in a large long-term observational study. We address time-dependent confounding by using marginal structural models and provide a non-technical practical demonstration of how to implement this type of modeling.

**Methods:**

Data is from 639 elderly individuals ascertained in the European multi-center Leukoaraiosis and Disability study (LADIS), followed-up yearly over a period of three years.

We estimated the effect of self-reported physical activity on the probability to transit to instrumental disability in the presence of a large set of potential confounders.

We compare the results of “conventional” modeling approaches to those estimated using marginal structural models, highlighting discrepancies.

**Results:**

A “conventional” Cox-regression-like adjustment for salient baseline confounders signals a significant risk reduction under physical activity for later instrumental disability (OR 0.62, 95% CI 0.44–0.90). However, given MSM estimation, the effect is attenuated towards null (OR 1.00, 95% CI 0.57–1.76).

**Conclusions:**

Contrary to most reports, we did not find that physical activity in older people prevents future instrumental disability, when taking time-dependent confounding into account. This result may be due to the characteristics our particular study population. It is, however, also conceivable that previous evidence neglected the effect of this type of bias.

We suggest that analysts of longitudinal observational studies consider marginal structural models as a further modeling approach.

**Electronic supplementary material:**

The online version of this article (10.1186/s12877-017-0657-3) contains supplementary material, which is available to authorized users.

## Background

Physical inactivity has been attributed to an increased burden of disease [1]. Conversely, subjects engaging even in small, leisure amounts of physical activity show reduced morbidity attributed to a wide variety of conditions including cancer or diabetes in comparison to those remaining inactive [2]. This is also seen for more acute conditions such as ischemic stroke [3], but not conclusively for pathophysiologically similar etiologies such as ischemic cerebral white matter pathology [4], highlighting heterogeneity of effects. These primary preventive benefits are not limited to manifest disease, but seem to be related in a more general sense to the maintenance of well-being or sustainment of activities of daily living.

Notwithstanding the fact that these beneficial results of physical activity are replicable across different settings and populations including older people, are supported by well designed and at times very large prospective cohort studies – and are pathophysiologically plausible, next to easily appealing to common sense – they are observations only and have not been conclusively derived by experiment [5]. Therefore claims that physical activity *causally* prevents disability must be interpreted with caution. Unfortunately – for all practical purposes – randomizing populations to be physically active over longer periods of time and comparing them to others that were instructed to remain inactive is infeasible.

As such, one is left with observation and perilous confounding. It is therefore important to conceptualize the mechanics of long term observational studies such as those investigating the effects of physical activity on disability in older people – being aware of potential bias.

**Fig. 1:**
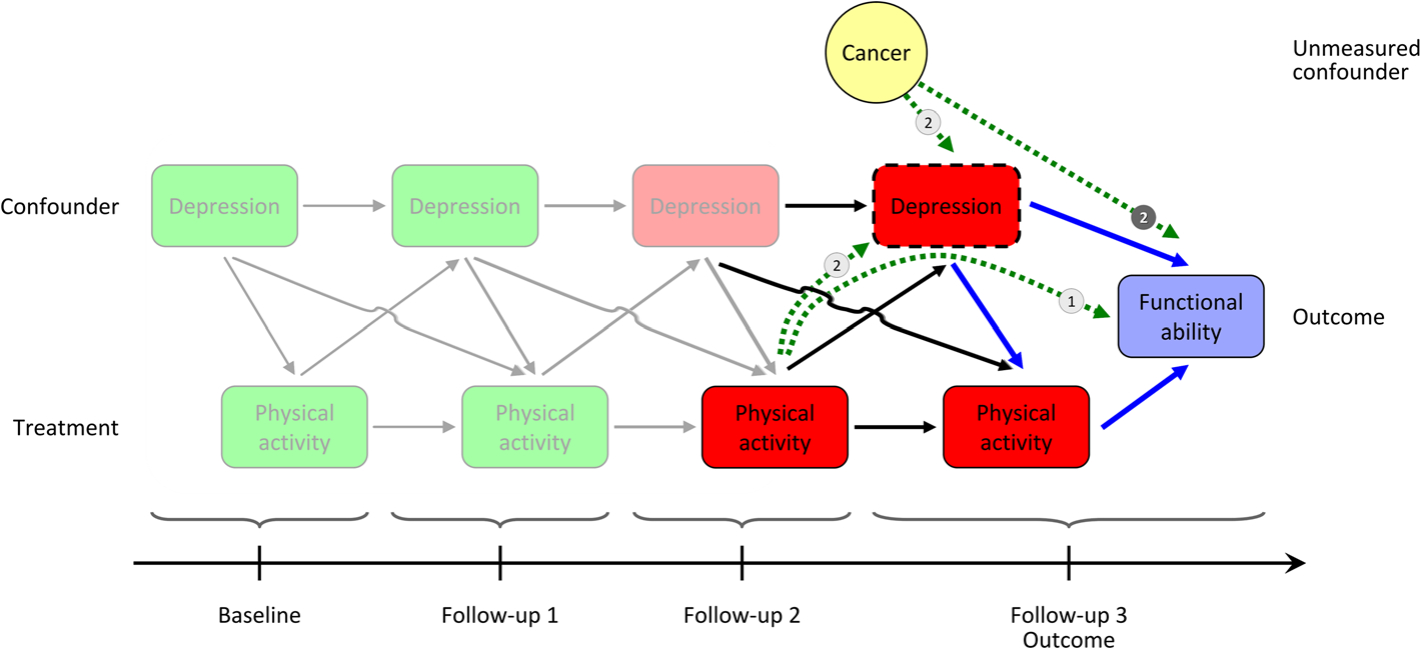
Time-dependent treatment and confounding: How does it come about? This schematic causal diagram illustrates the treatment with “physical activity” on the outcome status of “functional ability” in a hypothetical longitudinal study, which includes follow-up measurements of both time-varying status of treatment and confounders. For details in respect to the figure, see the heading “Time-dependent treatment and confounding: How does it come about?” in the “Methods” section

From the outset, two aspects seem highly improbable: That physical activity, as a form of treatment, remains at one and the same level per individual throughout the period of observation, and that confounders will be stable across time (see Fig. 1) – therefore, there is in fact, an individual varying *history* for both variables. For example, subjects reporting physical activity and good health at baseline may become depressed (a potential confounder) at some point; they may then also be less likely to continue being active (the time-varying state of the confounder therefore influences the propensity of future treatment). At a later time when mood has improved, they may start exercising to keep spirits up and with the intention to prevent disability.

In such a setting both treatment and confounding is not stable over time, but rather time-varying, the effects of both characteristics on outcome intermingle. At first sight, adjusting for these associations (e.g. by including these time-varying variables in a “conventional” Cox-regression model) should disentangle specific effects. Regrettably, it has been shown that given time-varying treatment in the presence of time-varying confounding “conventional” adjustment introduces a form of selection bias [6], distorting estimates – see Sterne et al. [7] or Gerhard et al. [8] for practical examples of how severe this can be.

Fortunately, there is a relatively new class of statistical models that allow for this type of estimation called marginal structural models (MSMs) – which are now used more often in applied research with data derived from observation. The question if physical activity in older people prevents downstream disability has previously not been analyzed taking time-varying confounding into account. Here we do so with data from the European multi-center Leukoaraiosis and Disability study (LADIS), which included 639 elderly individuals, with planned yearly follow-ups for at least three years after baseline cerebral magnetic resonance imaging (MRI), surveyed on a large set of clinical parameters, most of which have the potential to act as time-varying confounders [9, 10]. We also look at if cerebral white matter pathology modifies the effects. Moreover, we provide a non-technical practical demonstration of how to implement this type of modeling using standard statistical software in the Supporting information (which are found in the Additional file 1), discussing benefits and caveats.

MSMs are models that estimate the unconditional (meaning not depending on covariates; also called “marginal”) mean counterfactual outcomes (also called “structural”) – given certain prerequisites – and can be used for causal inference.

Recall: Given experiment, groups randomized to one or another treatment are unconditionally exchangeable, meaning that the characteristics of individuals in one treatment group are (theoretically) identical to those of the other. This is one essential precondition for causation: Although it is impossible (and therefore *counter to fact*) to observe an alternative outcome on an individual level even when there is randomization, had that particular individual received any treatment other than the actual treatment, exchangeability is viable on the group level.

Marginal structural models *emulate* exchangeability in observational studies, given (the, however, unrealistic assumption) that one has unbiased information on *all* confounders. This becomes possible by *weighting* the parameters of an outcome model (e.g. a “conventional” Cox-regression model) by the inverse conditional probability of an individual having received his or her own treatment (or *treatment history)*, given the individual levels of his or her set of confounders (or *confounder history)*. Hernán and Robins present a well readable introduction to why this works [11]; we also refer to the Supporting information of this manuscript. (The estimated weight is called the “inverse probability weight” and is abbreviated as “IPW”; these are similar to albeit not interchangeable with propensity scores.)

By auspicious coincidence (which in actual fact it is not), MSMs can deal with bias introduced by these time-dependent associations (in short: time-dependent confounding). The IPW is a weight that incorporates an individual’s treatment *history*, given his or her confounder *history.* (For the rest of the paper “treatment” (being actively exposed) will be seen as being interchangeable with “exposure” (being passively exposed, as would be the case for self-reported physical activity).)

MSMs therefore provide a way to analyze longitudinal observational studies that include repeated measurements of treatment and confounding variables, potentially emulating randomized trials. Given that certain prerequisites are fulfilled (some of these being difficult, if not impossible to surmount – see the “Discussion” section), estimates can be interpreted as expressing causal effects.

## Methods

See Pantoni et al. for more details on recruitment and other details of the European multi-center LADIS study protocol [9]. LADIS was planned to test the association of risk factors, foremost those related to cerebral imaging related pathology, with possible downstream transition to instrumental disability. Written informed consent was required from the participant or, if not possible, by his/her legal guardian. Ethical approval for the LADIS study was given by all local ethics committees.

Importantly, older people (65 to 84 years of age) were recruited only if they were free of instrumental disability at baseline and cerebral imaging using MRI showed at least some degree of age-related white matter change (ARWMC). 639 subjects were seen in person at one of 11 recruitment sites on a yearly basis thereafter (telephone interviews were performed if this was not possible), resulting in a maximum of three contacts post-baseline.

Note that LADIS is not a “true” population based study, but rather a longitudinal cohort comprised mainly of subjects that received initial cerebral imaging as part of work-up for numerous complaints (e.G. *minor* neurological or psychiatric complaints in the domains cognition or mood, not limiting instrumental functioning). Subjects will therefore most likely not be representative of a community dwelling population.

### Treatment, outcome, confounders

The LADIS protocol defined physical activity as being “present” if an individual self-reported at least 30 min of physical activity on at least three days of a given week [12]. Note following important constraints. 1.) The status of physical activity was enquired in respect to the time period from a given interview to the *previous* one. As such, it was intended to be an average measure, usually covering a period of one year. This introduces recall bias, measurement error and inconsistency in the definition of “physical activity”. As pointed out above, although “physical activity” is a passive exposure, we interpret it for the purposes of this study as being analogous to a form of treatment. (When “physical activity” is put in quotation marks it implies this study-specific treatment in contrast to physical activity as such.) 2.) There was no external validation of the measure. This introduces measurement error.

Informants rated the subjects’ instrumental activities of daily living abilities at baseline and on each follow-up on an eight item scale [13]. The outcome “transition to instrumental disability” was reached if a subject scored more than “no impairment” on at least two of eight items. Informants were asked to approximate the time-point of change. Again, both recall bias and measurement error are potentially introduced.

A confounder is considered to be a variable that is a common cause for both treatment and outcome. Whereas this definition may be verifiable in a simple non-time-varying setting with only a limited number of variables, it becomes difficult to empirically select salient confounding factors from a broad range of information in time-varying scenarios: A variable may confound at one time-point and not at another, or a variable may become a confounder only in concert with other selected variables. Referencing Hernán et al. [14], expert knowledge may need to influence the decision if or if not a variable is indeed defined as a confounder. Table 1 shows those variables that we considered a priori to be confounders [10, 15] – selected from over 50 covariates available in the original LADIS study database.

**Table 1:** Variable characteristics and model selection

	Baseline characteristics	Characteristic is missing^a^	Characteristic is time-varying^b^	Characteristic's association withthe propensity for physical activity (treatment)^c^	*p*	Characteristic's association with transition to disability (outcome)^d^	*p*	Association with treatment and/or outcome^e^	Variables included in final modeling
Age at baseline (mean (SD))	74.1 (5.0)	0.0	NA	1.0 (1.0 - 1.0)	0.190	1.1 (1.0 - 1.1)	<0.001	outcome only	x
Gender (% female)	54.8	0.0	NA	1.2 (0.9 - 1.6)	0.360	0.9 (0.7 - 1.1)	0.250	none	
Level of education (%)^f^	51.3	0.2	NA	2.0 (1.5 - 2.7)	<0.001	0.5 (0.4 - 0.6)	<0.001	both	x
Marital status (%)		22.4	6.6		0.016		0.032	both	x
Single	4.9			reference		reference			
Married or partnership	63.1			2.1 (1.0 - 4.3)	0.055	0.7 (0.4 - 1.2)	0.220		
Widowed	27.3			1.4 (0.7 - 3.1)	0.340	1.0 (0.6 - 1.9)	0.960		
Separated	4.7			3.8 (1.4 - 10.3)	0.008	0.5 (0.2 - 1.3)	0.160		
White matter pathology (%)^f^		0.0	NA		<0.001		<0.001	both	x
Mild	44.4			reference		reference			
Moderate	30.9			0.9 (0.6 - 1.2)	0.420	1.6 (1.1 - 2.3)	0.009		
Severe	24.7			0.5 (0.3 - 0.7)	<0.001	3.2 (2.3 - 4.4)	<0.001		
Stroke (%)^g,f^	7.2	0.0	NA	1.0 (0.5 - 1.8)	1.000	1.7 (1.1 - 2.6)	0.021	outcome only	x
Alcohol consumption (%)^f^	55.6	0.3	NA	1.1 (0.8 - 1.5)	0.670	0.9 (0.7 - 1.1)	0.320	none	
Atrial fibrillation (%)^g^	7.5	23.0	5.3	1.1 (0.6 - 1.8)	0.820	1.7 (1.1 - 2.5)	0.013	outcome only	x
Angina pectoris (%)^g^	15.4	23.2	12.1	0.7 (0.5 - 1.0)	0.050	1.5 (1.0 - 2.1)	0.040	both	x
Anxiety or depressed mood (%)^f,g^	36.8	23.6	32.6	0.4 (0.3 - 0.5)	<0.001	1.8 (1.4 - 2.4)	<0.001	both	x
Body mass index (kg/m^2^; mean(SD))^f^	26.2 (4.2)	34.1	30.4		0.060		0.266	treatment only	
Middle tertile (range)	24.2 - 27.3			reference		reference			
First tertile (range)	16.4 - 24.2			1.2 (0.9 - 1.8)	0.250	1.3 (0.9 - 1.8)	0.140		
Last tertile (range)	27.3 - 47.8			0.8 (0.6 - 1.1)	0.150	1.3 (0.9 - 1.8)	0.190		
Chronic pain (%)^g^	33.0	23.0	27.2	0.4 (0.3 - 0.5)	<0.001	1.2 (0.9 - 1.5)	0.300	treatment only	
Complaints of gait disturbance (%)^g^	40.8	23.5	30.8	0.3 (0.2 - 0.4)	<0.001	2.6 (1.9 - 3.4)	<0.001	both	x
History of falls (%)^g^	28.6	22.7	39.6	0.6 (0.4 - 0.8)	<0.001	1.9 (1.4 - 2.5)	<0.001	both	x
Major depressive episode (%)^f,g^	6.1	22.5	9.1	0.6 (0.3 - 1.2)	0.130	2.9 (1.9 - 4.5)	<0.001	outcome only	x
MMSE (%)^f^	11.8	23.5	14.4	0.5 (0.3 - 0.7)	<0.001	4.1 (3.1 - 5.5)	<0.001	both	x
Osteoarthritis (%)^g^	28.1	23.0	22.7	0.7 (0.5 - 1.0)	0.032	0.8 (0.6 - 1.1)	0.170	treatment only	
Peripheral vascular disease (%)^g^	7.2	24.1	6.6	0.5 (0.3 - 0.8)	0.006	1.2 (0.7 - 1.9)	0.510	treatment only	
Smoking (%)^g^	45.8	0.2	NA	1.0 (0.7 - 1.3)	0.870	0.9 (0.7 - 1.2)	0.370	none	
Syncopal episodes (%)^g^	16.6	23.5	17.7	0.5 (0.3 - 1.0)	0.036	2.4 (1.5 - 3.9)	<0.001	both	x

NA Not applicable as variables were recorded as non-time-varying

^a^Percentage of individuals reporting at least one missing data point per time-varying characteristic or completely missing in non-time-varying characteristics given a complete cohort N of 639 individuals

^b^Percentage of individuals reporting time-varying data per characteristic (e.g. “Marital status” changed at least once over the course of time in 6.6% of the cohort)

^c^Odds ratio for the association between “physical activity” and characteristic over all time-points (taking individual-specific clustering into account); higher ratios indicate more likely to be “physically active” given the characteristic or per unit increase in characteristic

^d^Hazard ratio for the association between “physical activity” and characteristic; higher ratios indicate more likely to transit to disability given the characteristic or per unit increase in characteristic

^e^Associations are highlighted; note that an association is arbitrarily defined as being “relevant” if the univariate regression model is significant to *p* < 0.1 (see the Supporting information for details of why this cut-off is chosen)

^f^Level of education: 10 years of education or less of formalized education (reference) vs. more education; Degree of ARWMC rated according to “Fazekas criteria”; Stroke: recorded stroke event independent of any severity post-baseline; Alcohol consumption: any alcohol consumption, including for the largest part casual drinking (reference) vs. none; Anxiety or depressed mood: self-reported; Body mass index: baseline tertiles - change is in respect to baseline. Major depressive episode: DSM-IV expert rating; MMSE: >24 points (reference) vs. worse performance

^g^Characteristics are not present (reference) vs. they are

### Time-dependent treatment and confounding: How does it come about?

Fig. 1 introduces the reader to the mechanics of time-varying treatment in the presence of time-varying confounding. This is illustrated as a schematic causal diagram – details of which are explained below – of the expected effect of treatment with “physical activity” on the status of “functional ability” (i.e. the outcome would be disability) in a hypothetical longitudinal study (but closely analogous to our study), which includes follow-ups allowing measurement of the status of treatment and confounders (such as the history of depression).

Unfortunately, in this particular example, the study plan forgot to include a further confounder, namely the history of cancer – and as such, it remained unmeasured.

The diagram implies that for this specific a subcohort of the population, treatment with “physical activity” was present (green fillings) at baseline and the first follow-up, but subjects remained physically inactive on the second and third follow-ups (red fillings). Outcome was measured at the third follow-up. “Depression” was absent (green fillings) at baseline and time-point one; “depression” was present at the subsequent follow-ups (red fillings).

All solid arrows highlight potential associations (and their direction), including lagged effects (higher order lags are not shown; also, the unmeasured confounder “cancer” is measured throughout, but only shown on the last follow-up).

Take special note of the boxes connected via the blue arrows to the right. This constellation implies that “depression” (dashed box at follow-up 3) acts as a confounder on the causal pathway of the effect of treatment with “physical activity” on outcome; treatment and outcome both have a common cause, namely “depression” (arrows point from “depression” to the treatment and outcome). In a non-time-varying setting, adjusting via conventional methods for (i.e. conditioning on) the confounder “depression” would result in an unconfounded (albeit conditional) estimate of the effect of interest.

Unfortunately, in a time-varying setting bias may instead be introduced by adjusting for “depression” (again – dashed box at time-point “follow-up 3”) via following mechanisms:

Firstly, the treatment “physical activity” at follow-up 2 is associated with the confounder “depression” at follow-up 3 – e.g. being previously “physically active” may prevent present “depression”. Also, present “depression” will influence the probability of developing future disability. If one expects that most of the effect of “physical activity” on outcome is mediated through the presence or absence of “depression” (this pathway is highlighted by the dotted green line with the label ①) – “depression” therefore not only being a confounder, but also acting as an intermediate variable – adjusting for “depression” would block this pathway, introducing bias.

Secondly, assume for a moment that in true fact there is no direct causal effect of physical activity at follow-up 2 on outcome (by ignoring the arrows between “physical activity” at follow-ups 2 and 3, and from there to the outcome). But, “physical activity” at follow-up 2 affects the status of the variable “depression” at follow-up 3 as does the unmeasured confounder “cancer” (latter connection highlighted by the dotted green arrow with the label ②). In other words, they share a common effect. Also, the unmeasured confounder “cancer” is associated with the outcome (dotted green arrow with the label ➋). By adjusting for the confounder “depression” via conventional methods, one is in fact inducing an artificial association between physical activity at follow-up 2 and outcome, where in reality there is none. This equates to a selection bias of the variable “physical activity” (at follow-up 2; see Hernán et al. [6] for a thorough explanation and Cole et al. [16] for an easily understandable example).

### Marginal structural models and statistics

Time-dependent confounding (and time-varying treatment) can be dealt with using marginal structural models (MSM). We refer the reader to the Supporting information (these are found in the Additional file 1) for a practical introduction on how marginal structural models can be estimated generically, including further information on caveats. A summary of estimation steps used in this specific study is presented below. Information on each of these steps is cross-referenced in the Supporting information under the heading “Marginal structural models and statistics: Detailed methods”.

For standard authoritative publications on MSMs see Robins and colleagues [17, 18]. See Fig. 1 and the figure in the Supporting information for schematics of the underlying problem MSMs solve.

In essence estimation is a two-step process, requiring first the calculation of an individual and time-point specific weight (the IPW); this is the treatment model. Secondly, one then performs the analysis of treatment on outcome, weighted by the IPW – this it the outcome model. Here we estimate a “marginal structural model for survival analysis” using “pooled logistic regression” weighted by the IPW, given a dichotomous treatment (see the Supporting information for other model specifications).

In the study presented here, variables included in the estimation of the IPW (i.e. the treatment model) were those that were considered to be potential confounders (i.e. associated with both treatment and outcome) or were associated with outcome only (which may reduce bias further – see the Supporting information why this is so) – if the univariate regression model was significant to *p* < 0.1; for sake of simplicity, latter variables are also subsumed under the term “potential confounders” (see Table 1’s last column marking these variables). These included both static (e.g. “level of education” or “white matter pathology” at baseline) and time-varying variables (e.g. “history of falls”). We also included baseline values and lags (i.e. data of the previous follow-up time-point) of time-varying variables (potential confounders *and* the treatment variable). Moreover, the time variable as quarters of a year from baseline and a spline function of the time variable were entered as linear terms. The treatment “physical activity” was regressed on these variables using logistic regression, followed by the prediction of “observed” treatment. The individual-specific “cumulative propensity” was calculated thereafter for each observed time-point. This is the denominator of the weight. The numerator was calculated analogously, however, it included static variables, the baseline variables for time-varying variables and the treatment history only (i.e. omitting follow-up data for the potentially confounding variables and their lags); and the time variable and its spline. The (row-wise) quotient of numerators and denominators is then the so-called “stabilized” (inverse) treatment weight.

Multiplying this weight by a further stabilized “censoring” weight leads to the stabilized IPW. We truncated the stabilized weight at its 1st and 99th percentiles.

The outcome model is a logistic regression model where the outcome “transition to disability” is regressed on the treatment “physical activity”, and the time variable and its spline, weighted by the IPW. Although a MSM is theoretically an unconditional model, further predictors had to be added to the estimation due to the way the IPW was calculated. These were those variables used in the estimation of the numerator (excluding treatment history).

To demonstrate how different adjustment strategies for potential confounders affect estimation of disability free survival given physical activity, the results of the marginal structural model was compared to an unadjusted pooled logistic regression model, a baseline adjusted pooled logistic regression model and a pooled logistic regression model that includes time-varying values for treatment and confounders. Note that pooled logistic regression was used instead of “conventional” Cox-regression models to analyze survival given technical constraints in the way weights can be handled in most statistical software packages. Pooled logistic regression approximates proportional hazard models.

## Results

Table 1 highlights the characteristics of those variables deemed to be confounders of the putative causal association between “physical activity” and transition to disability. It shows that not all of these variables are in fact confounders. For example, smoking is neither associated with self-reported “physically activity” nor is it associated with a transition to disability. Other variables, such as a history of osteoarthritis, do in fact significantly hamper the ability to be “physically active”, but are unassociated with outcome.

Most variables are non-constant over time. For example, marital status changes in 6.6% of the cohort during the course of the study; the status self-reported gait disturbance is highly dynamic, changing in 30.8% of the individuals at least once on repeated questioning. Self-reported “physical activity” is non-constant in 27.7% of the cohort (46.2% report constant activity, 26.2% constant inactivity). 32.9% of the cohort transited to instrumental disability, on average 1¾ years after baseline (median 1½ years, IQR 1–2 ¼ years).

**Fig. 2:**
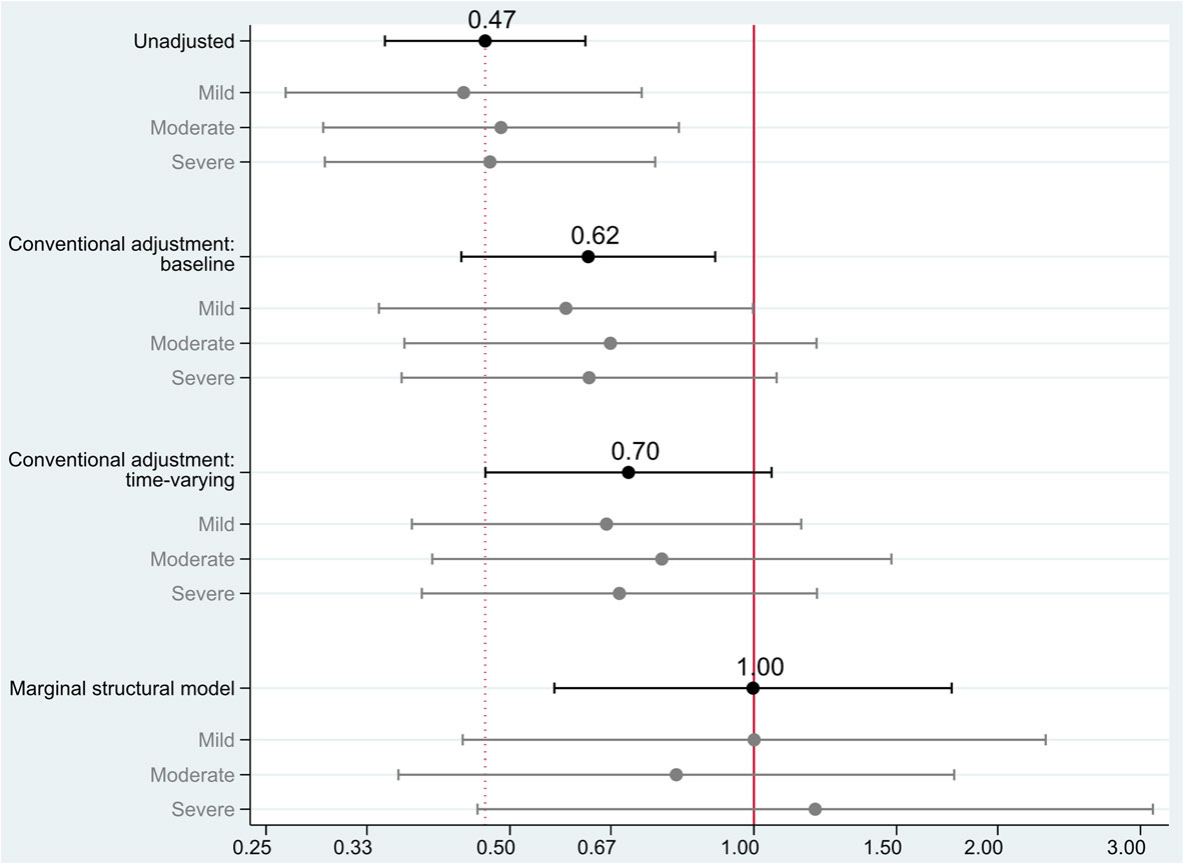
The effect of “physical activity” on the risk of transition to disability. The unadjusted model and models marked “conventional adjustment” estimate the effect using a pooled logistic regression model – which is comparable to a Cox-regression analysis – with different degrees of adjustment as indicated. Estimates are therefore not the customary hazard ratios, but are instead expressed as odds ratios. The labels “Mild”, “Moderate” and “Severe” refer to estimates given different degrees of age-related white matter lesions

Fig. 2 summarizes the effect of “physical activity” on the transition to instrumental disability given different modeling approaches.

If potential confounders are simply ignored, the treatment “physical activity” indicates a substantial protective effect on outcome: An average individual that had reported being physically active at least 30 min on at least three days a week would have approximately 50% less odds of transiting to instrumental disability over the following quarter of a year (odds ratio 0.47, 95% CI 0.35–0.62, *p* < 0.001).

Clearly, this is a limited interpretation as it would ignore the effect of variables affecting treatment and outcome. Adjusting for these variables at baseline, reduces the predicted protective effect that “physical activity” might have on preventing disability (odds ratio 0.62, 95% CI 0.44–0.90, *p* = 0.011).

If one also considers the time-varying data structure using a “conventional” Cox-regression-like adjustment (but not addressing time-varying confounding), the association is attenuated further: Though the point estimate still indicates a protective effect, variability increases, rendering an overall insignificant contribution (odds ratio 0.70, 95%CI 0.47–1.05, *p* = 0.086).

Accounting for selection-bias induced by the time-varying effects of treatment and confounding using a marginal structural model approach – theoretically modeling a population based effect of physical activity on outcome in analogy to the mechanics of a randomized controlled trial – the effect of physical activity becomes equivocal (odds ratio 1.00, 95% CI 0.57–1.76, *p* = 1.0).

Of further interest was the question if the baseline variable “white matter pathology” (i.e. ARWMC) modified the effect. In other words: Do individuals with a specific lesion load benefit more from “physical activity” than others? All models’ point-estimates other than the MSM indicate that patients with either mild or severe degrees of ARWMC fare better given “physical activity” in comparison to those with moderate lesion load. This pattern is reversed in the marginal structural model estimation. Here patients with moderate white matter pathology seem to profit most from persistent “physical activity”, albeit given large variability in the estimate. None of these comparisons, however, reach statistical significance.

## Discussion

Firstly, it appeals to common sense that being physically active is beneficial on one’s future health.

However, it is, secondly, equally plausible that there is an association between being presently able to perform physical activities and the status of presently being healthy (and the contrary).

Moreover, thirdly, numerous other risk factors will likely influence the risk of future disability.

The results of this study give some support for the second and third hypotheses: many factors influence an individual’s propensity to report being physically active – such as having chronic pain, being afflicted by osteoarthritis or peripheral vascular disease. A major depressive episode, incident stroke and – importantly – age itself are all risk factors promoting a disabling outcome. Numerous variables influence both the likelihood of treatment and outcome (i.e. representing confounding), having cognitive impairment, for example.

At first sight there also is support for the first notion above: Ignoring other possible influences, contemporary “physical activity” very significantly prevents future instrumental disability (the odds of experiencing disability is 50% less for the next quarter of a year when being “physically active”). This is in line with the results summarized across nine studies in a recent meta-analysis [19]. The authors found a pooled odds ratio of 0.51 given previous “medium” or “high” levels of physical activity (the self-reported level of positive physical activity in the present study theoretically lies somewhere between “medium” and “high”). This effect was estimated given various degrees of adjustment in individual studies for demographic variables, lifestyle factors, functional limitations and health status. Note, however, that the summary estimate included only baseline adjusted data, as is common in many prospective cohort studies: An individual is seen thoroughly once at study entry, acquiring the full breadth of information; outcome (but not further variables) is then ascertained through repeated screening thereafter. Therefore, time-varying treatment and confounding will not have been adequately acknowledged.

There are some notable exceptions in the literature. For example, Liao and co-authors analyzed the effect of healthy behaviors including regular exercise in four waves over a ten year period [20]. When adjusting for time-varying health status (i.e. confounding; but ignoring time-varying treatment status) using a typical Cox-regression modeling approach, exercise reduced the risk of functional disability by 34% – resembling the estimate from our third model.

Keysor pointed out that the majority of observational (non-experimental) prospective cohorts highlight a beneficial effect of physical activity on downstream disability; *experimental evidence*, however, is far from conclusive, with most studies not showing that physical activity is *causally* preventive [5]. This notwithstanding, randomized trials in older populations testing the effect of less comprehensive therapies such as muscle strengthening or balance training on change in ability in simple motor activities in the short term do prove to be effective [21]. These results, however, do not readily transport to more complex measures of disability [22] and may confer benefit only in certain, already disabled populations. Most authors, moreover, underline the scarcity of available studies, perhaps reflecting the difficulty in carrying out complex experiments with long periods of follow-up [23], and reverberate difficulties in comparability with heterogeneous definitions of interventions and outcomes [24].

*Theoretically*, MSMs provide unconditional population causal effect estimates from observational studies – these can therefore *potentially* be interpreted analogously to results from randomized controlled trials. Marginal structural models take into account that treatment is dependent on prior treatment and on prior confounder status, both being potentially associated with outcome.

Doing so attenuates our previously estimated preventive effects using “conventional” modeling towards null: Taking into account that an individual’s time-varying health status is associated with future treatment (second hypothesis above), and the health status also predicts outcome (third hypothesis), may explain this non-beneficial (but also non-detrimental) effect (refuting the first hypothesis above). Self-reported “physical activity” as such is not sufficient to prevent transition to instrumental disability (*in the data analyzed here*).

### Constraints of marginal structural models

Albeit the useful characteristics of MSMs allowing some approximation of treatment effects in the observational setting otherwise not accessible in experiments, we *cannot* argue conclusively against a beneficial causal effect of physical activity on the advent of instrumental disability. The results from marginal structural model analysis must be interpreted with caution. There are several reasons for this. Next to the lack of external validity – extrapolation of the results is inherently limited to populations “like” the one ascertained in the LADIS study – there are numerous analytical constraints (next to numerous forms of bias as pointed out in the “Methods” section and in the Supporting information).

Firstly, MSMs are only valid if both the treatment and the outcome models are specified correctly (Note that this is an equal pre-condition for “conventional” regression models.). There is no way “proving” that we have succeeded here. Correct model specification would mean, for example, that we are certain about how the variables included “relate” to each other. For example, though neither alcohol nor smoking are associated significantly with either the propensity for “physical activity” or future disability, their interaction may well be; we, however, did not test all interactions. Or: We don’t know if treatment conveys a dichotomous yes/no effect (which it surely will not). However, we are modeling one here. Also, erroneously including potentially non-confounding variables will re-introduce bias and reduce precision [25].

Secondly, it must be guaranteed that at each level of a confounder there is data available for exposed and unexposed individuals. This prerequisite, also called “positivity”, is nearly impossible to fulfill in high-dimensional data, moreover given many continuous variables (see the Supporting information for a way to colloquially check validity).

Thirdly – as a prerequisite for correct model specification – all potential confounders must have been measured and without error at that; latter condition also applies for both the treatment and outcome variables (e.g. we could not validly quantify self-reported “physical activity”). It is quite probable, if not certain, that we have missed informative data and are left with residual confounding. Other factors potentially associated with treatment and outcome – such as infections, cancer or other indicators of health status, and a more precise measurement of psychosocial determinants were unavailable [26].

Fourthly, the confidence intervals estimated using the MSMs are wider than under conventional adjustment and may indicate that although one is now adequately accounting for bias, the estimation of the IPW remains inefficient. In short: Although the sample size is quite large, it may still be too small to detect real effects given this modeling approach.

Finally, though less relevant (but not irrelevant; see [27]) for the aspect of estimating unbiased and unconditional effects: For marginal structural models to be valid in respect to providing truly causal estimates, the treatment – more specifically the causal contrast under investigation – must be “consistent”. Consider the following: Our causal contrast was self-reported “physical activity” above a certain cut-off (see the “Methods” section) versus not being so. “Physical activity” could mean that an individual was active playing golf three times a week, walking vigorously daily or doing 35 min of upper-body exercise on Fridays, Saturdays and Sundays. Given this heterogeneity, we are unable to define a contrast narrow enough to specify a potential causal mechanism [28]. Would one plan an experiment with the randomized intervention being: “Indiscriminately perform physical activity on at least three days a week for at least 30 minutes or more.”? Most likely one would expect a much more specific treatment plan.

### Consistency of treatment and causal interpretation

There is another aspect relevant for a valid causal interpretation. LADIS – the study from which the data was drawn – was designed to investigate risk factors associated with the development of ARWMC and clinical features of individuals in respect to different degrees of pathology. It might seem tempting to investigate the potential “causal effect” of white matter pathology (i.e. the exposure or, synonymously, treatment) on the transition to disability (i.e. the outcome) – knowing that there is a well documented “association” [10] – using MSMs, given their desirable properties. For example, one might want to construct the “causal contrast” “mild lesion load” versus “severe lesion load”. Though analytical feasible, such an attempt will not lead to plausible conclusions. The reason is two-fold. Firstly, as in the example above: We may now have a consistent definition of the causal contrast (i.e. we know the exact “amount” of pathology), but we are completely agnostic as to the mechanism that causes this pathology – and in turn may be at least partially (also) causing disability. Is it diabetes in one individual? Is it hypertension in another? Is it genetics? So if we contrast “mild” with “severe” pathology, we do not know if it is a contrast of white pathology due to diabetes or due to hypertension? The causal pathway to disability may differ depending on the underlying pathophysiology. Secondly – perhaps conceptually more important – contrary to the “physical activity” intervention, what are we to manipulate? There is no way to “treat” with “mild” versus “severe” white matter pathology. Causal inference generally becomes futile without the possibility of manipulation [29].

However, MSMs do allow us to study the potential association of non-time-varying static variables (e.g. baseline variables) on treatment and their effect on outcome. For example, our data suggests that, contrary to estimates ignoring time-varying bias using “conventional” regression, it may in fact be those individuals with “moderate” ARWMC that profit more from “physical activity” in contrast to those with “mild” or “severe” lesion load. Though perhaps pathophysiologically plausible – those with “moderate” pathology may still have more “brain reserve” than those “severely” affected (latter may perhaps also be more prone to injuries related to physical activity), those with “mild” pathology simply may not benefit from intervention – we have no evidence to refute the possibility that this pattern is nothing but a chance finding.

## Conclusion

Concluding, in situations where randomized controlled trials are infeasible as in the question at hand here, analyzing longitudinal observational data with repeated measurements of treatment and confounders, given otherwise uncontrollable bias introduced by time-varying associations, marginal structural models allow for a relatively easily implementable causal analysis – if pre-requisites are considered, their intricacies and potential fallacies understood.

## Additional file

Additional file 1: Supporting information. The Supporting information provides a non-technical introduction on how marginal structural models can be estimated generically. We provide a practical demonstration of how to implement this type of modeling using standard statistical software, discussing benefits and caveats. We include an additional figure that highlights the underpinnings of time-varying treatment, time-varying confounding and the inverse probability of treatment weight. The estimation steps presented in the main manuscript are cross-referenced in the Supporting information. (DOC 1488 kb)

## Abbreviations

ARWMC: Age-related white matter lesions
LADIS: Leukoaraiosis and disability study
MSM: Marginal structural models
