# Supplementary material for: Does being physically active prevent future disability in older people? Attenuated effects when taking time-dependent confounders into account
Source: BMC Geriatr. 2017 Dec 21;17:290. doi: 10.1186/s12877-017-0657-3 (PMC5740527; doi:10.1186/s12877-017-0657-3)
Supplement: Additional file 1: — Supporting information. The Supporting information provides a non-technical introduction on how marginal structural models can be estimated generically. We provide a practical demonstration of how to implement this type of modeling using standard statistical software, discussing benefits and caveats. We include an additional figure that highlights the underpinnings of time-varying treatment, time-varying confounding and the inverse probability of treatment weight. The estimation steps presented in the main manuscript are cross-referenced in the Supporting information. (DOC 1488 kb) [file 12877_2017_657_MOESM1_ESM.doc]

# Supporting information

## Does being physically active prevent future disability in older people? Attenuated effects when taking time-dependent confounders into account

Readers that have come in touch with original publications whose results have been derived using MSMs may want to understand the steps involved in their estimation without delving deeper into the statistical underpinnings involved. However, we find that more focused methodological or epidemiological skills are required for many tutorials published previously (see below for a selection). Here we hope to provide a non-technical introduction to the marginal structural model and how to estimate its parameters. The reader can “use” the data in the tables below to replicate the calculations using his or her own statistical software. We provide coding examples for Stata, SAS and R under the subheadings “Practical implementation”. (To our knowledge, there is to date no “point-and-click” software available to estimate (longitudinal, time-varying) marginal structural models (though R packages go a long way)).

Moreover, there are numerous potential caveats related to the *characteristics of the data* (variable selection, dealing with missings, censoring), *data preparation* and the *process of estimation*: the prediction of the inverse probability weight (i.e. the treatment model) and the specification of the outcome model. We explain these steps and highlight remedies. Also – although the study presented in the Main manuscript implemented a marginal structural model for survival analysis – one may be interested in estimating other outcomes or the effect of non-dichotomous treatment. We provide advice on how to use MSMs when dealing with alternative *model specifications*.

The final section headed “Marginal structural models and statistics: Detailed methods” provides further details on the estimation used in the Main manuscript.

## Table of contents

Data preparation 4

Process of estimation 8

I.) Treatment model 8

i.) Regression 9

ii.) Prediction 9

iii.) Cumulative propensity 10

iv.) Inverse probability weight 10

II.) Outcome model 12

III.) The final MSM 13

Characteristics of the data: Model specification 14

Caveat: Unstable inverse probability weight 14

a.) Stabilization 14

b.) Truncation 15

Caveat: Treatment model variables 15

a.) Selecting variables associated with treatment and/or outcome 17

b.) Time-invariant variables 17

Caveat: Missing data 19

Model specification: Modeling outcome other than survival; non-dichotomous treatment 20

Outcomes other than survival 20

Non-dichotomous treatment 20

Model specification: Modeling survival [22](#__RefHeading___Toc453602214)

Characteristics of the data: Censoring and other weights 23

Marginal structural models and statistics: Detailed methods 24

Data preparation; Missing data 24

Unstable inverse probability weight; Treatment model variables; Process of estimation – Step I 25

Process of estimation – Step II 28

Figure 29

References 32

## Data preparation

The dataset should be set up in long form (irrespective if one is interested in a survival analysis or other outcome model specification) – this means that there is one observation per subject per time point. E.g.:

*““Time- ““Time- “Baseline*

*varying” varying” or static Generic*

*treatment” confounder” variables” description*

Visit Physical Depression Depression Variable

ID date activity status at baseline Disability description

subject_ static_ Variable

id visit treatment confounder confounder outcome name

*...*

**2 baseline** NA NA **0** NA

**2 12 months 1 0** NA **0**

**2 24 months 0 0** NA **0**

**2 30 months 0 1** NA **1**

**3 baseline** NA NA **1** NA

**3 16 months 0 0** NA **0**

**3 24 months 1 1** NA **1**

*...*

*NA Not applicable*

Data set-up: Survival analysis with pooled logistic regression

If one is interested in estimating survival using the marginal structural modeling approach, one may need a further work-around not related to the underpinnings of MSM, but rather to the way most currently available software handles weights (i.e. instead of performing a marginal structural model for survival analysis using proportional hazard methodology as would be sensible, one must implement “pooled logistic regression”, requiring some data tweaking; see the heading “Model specification: Modeling survival” for an explanation why that is). This amounts to repeating observations. (If less restricted software becomes available or one is not interested in survival analysis, data preparation may ignore the instructions in the directly following paragraph, albeit data remains set up in long form.)

In detail: Say follow-up data for one subject (i.e. subject ID #2) was available at 12 months, at 24 months and the informant reported a transition to disability at 30 months. With “repeating observations” one means repeating the value of observations across variables for a specific number of times (i.e. extra rows of data) in respect to the time interval. One “natural” way to proceed would be to repeat the observations 12 months post-baseline 12 times, at 24 months 12 times (interval between the two follow-up dates is 12 months) and at 30 months 6 times. This set-up, however, is not tenable outside of very large datasets that would guarantee that real observations from at least some subjects occupy each and every time point. One must therefore often choose “coarser” time intervals – in above scenario, one quarter of a year, for example. In this case variables recorded at 12 months would be repeated for the first, second, third and fourth observation rows; rows five to eight would be repeat data of the ascertainment at 24 months; the remaining rows nine and ten would be copies of information recorded at the 30^th^ month.

Data set-up: Some generic instructions

Note that the baseline values of time-varying confounders are not included as separate observations, but rather as separate variables. If, for example, baseline depression status was negative (coded 0), that value would be repeated in all ten rows. Confounders such as depression post-baseline are considered to vary: If a subject remains without depression up to the second visit after 24 months, rows including this date and those before are coded 0. If the informant reported depression in the subject at 30 months, depression would be coded as 1 for that and previous quarters (up to the prior visit). The variable indicating outcome “disability” would remain coded negative (coded 0) up to row nine, switching to a positive code (coded 1) in row ten (If the individual does not switch, it remains negative (coded 0)). Values for static (i.e. eye color) or stochastically predictable variables such as age are replicated in the same way as baseline variables. Time-varying treatment (or exposure) variables are set up in the same way as time-varying confounders. If lags (i.e. values of time-varying variables at t-1 (or further back)) are to be included, one copies the values of previous observations to the current one; observations directly after baseline can include the values of the baseline data. See the following table.

*“Repetition of “Example*

*observations” for lags”*

Quarter

of a year Depression

running status

count at t-1

subject_ previous_ static_

id visit time treatment confounder confounder confounder outcome

*...*

2 1 1 0 0 0 0

2 2 1 0 0 0 0

2 3 1 0 0 0 0

**2 12m 4 1 0 0 0 0**

2 5 0 0 0 0 0

2 6 0 0 0 0 0

2 7 0 0 0 0 0

**2 24m 8 0 0 0 0 0**

2 9 0 1 0 0 0

**2 30m 10 0 1 0 0 1**

3 1 0 0 1 1 0

3 2 0 0 1 1 0

3 3 0 0 1 1 0

3 4 0 0 1 1 0

**3 16m 5 0 0 1 1 0**

3 6 1 1 0 1 0

3 7 1 1 0 1 0

**3 24m 8 1 1 0 1 1**

*...*

*Variable descriptions of variables introduced in the previous table are not shown again.*

*Arrows highlight backward repetition of data.*

Data set-up: Survival analysis caveats

There are two inherent problems with this set-up (but equally applicable to standard survival analysis) – introducing bias.

1.) One erroneously assumes that all variables remain stable in the time intervals between true visit dates. Depending on the characteristic, this may or may not be the case. Depression may in fact have also been present after 18 months and remitted 2 months later. Yet one assumes that this was not the case (even if a subject reported so). There is no remedy to this other than decreasing the time between follow-ups and adding further visits. Moreover, it is likely that when a subject reports e.g. not being depressive at 24 months, he/she will also not have been depressed immediately thereafter. However, one is agnostic on the subject’s status after a visit; rather, one erroneously extrapolates information available on the next visit (here now reporting “depression”) backwards.

2.) Causal analysis assumes that treatment precedes outcome (and confounder effects precede treatment). The data is ordered in such a way that treatment and confounders indeed precede outcome, and therefore implies an immediate effect one on the other. Clearly, real clinical information seldom provides for such mechanistic sequence. Also, confounder effects are not sequentially placed prior to treatment changes.

## Process of estimation

In essence estimation is a two-step process, a *treatment model* followed by an *outcome model*. A schematic of what is meant by time-varying treatment and time-varying confounding is depicted in the Figure at the end of this text. The table below further highlights the steps involved in calculation.

Note that standard statistical software (i.e. Stata, SAS, SPSS) does not provide for fool-proof automated analysis. However, there is a good step-by-step tutorial with code published for Stata [1]; coding examples are also found in the supplement of a recent more technical paper [2]. Moreover, a versatile public-domain macro is available for SAS [3]; also, two very thorough reports – readable also for users of other software – provide guidance [4,5]. SPSS in its basic installation does not allow the use of the type of weights needed to operationalize MSMs and is therefore not discussed further. R, on the other hand, provides more complete coverage: The package IPW estimates inverse probability weights [6] and the package LTMLE allows a complete marginal structural model analysis [7].

**I.) “Treatment model”**

Generate weights (the *inverse probability of treatment weight* (IPTW)) that reflect an individual’s *cumulative propensity* (i.e. probability) of having received his or her *observed* treatment at a given time-point, given previous treatment and confounder *histories* (Note that in the Main manuscript we referred to this weight as the inverse probability weight (IPW). While foremost incorporating the inverse probability of treatment, the IPW may also include further weights that reflect censoring or mechanisms describing missingness – see below). This somewhat unwieldy definition translates into following practical implementation (there are some coding examples below):

**i.) Regress the “observed” treatment (dependent variable) on treatment and confounder “histories” (independent variables)**

With “history” one means those variables that might have influenced the state of treatment at a given time-point. More precisely, the state of those variables measured *before* the time-point at which treatment was measured. Colloquially this means including as independent variables both a lag of the treatment variable (e.g. the value at t-1), and present and past states of confounding variables; how many lags back to include depends on the researcher’s hypothesis. (Note that in the practical example below, a static variable is included, namely the status of depression at baseline. Doing so departs from latter instructions for a prototypical MSM set-up. In practice, however, one does include numerous other variables as explained under the heading “Caveat: Treatment model variables” below.). Thereafter,

**ii.) predict the probability of “observed” treatment**

Prediction uses the estimated parameters of the variables included above to predict a model-specific individual probability of *positive* treatment. In above data this would mean: “Probability of having been physically active”. If, however, an individual in fact had been physically *inactive*, his/her observed treatment status would have been *negative* (or correctly: coded 0). In this case “true” treatment was therefore actually “no treatment”. Calculation of IPTWs necessitates the true, i.e. observed treatment probability. As statistical software is agnostic in respect to what a “true” treatment state is – it simply gives you the predicted probability of positive treatment – one has to subtract the predicted probability in those cases where observed treatment was in fact “no treatment” (i.e. “no treatment” *is the* treatment) from 1. See Fewell et al. for a coding “trick” when implementing the above in statistical software [1].

* Practical implementation

Commonly, in the case of dichotomous treatment procedures i.) is performed by logistic regression, followed by ii.) a “prediction” command for the estimated probabilities (see the heading “Modeling things other than survival; non-dichotomous treatment” for examples of different outcome and treatment forms):

Stata:
 logistic treatment previous_treatment confounders previous_confounders
 *followed by* predict propensity, pr

SAS:
 proc logistic {…} model treatment=previous_treatment confounders previous_confounders {…} output predicted=propensity

R (when not using LTMLE package):
 {…} <- glm(treatment ~ previous_treatment confounders previous_confounders, {…}, family = “binomial”)
 *followed by* {…} <- predict({…}, type=”response”).

**iii.) Calculate “cumulative propensity”**

Cumulative propensity in this context simply implies that the predicted probability of “observed” treatment for an individual at time-point t (i.e. the output “propensity” above) is dependent on the set of variables used to calculate that probability *and* the predicted probability at time-point t-1. In practice, one consecutively multiplies an individual’s present predicted propensity with their previous one. Finally,

**iv.) generate the “inverse probability of treatment weight” (IPTW)**

The IPTW is calculated by dividing the cumulative propensity for each time-point (i.e. this then is the *denominator*) by 1 (i.e. the *numerator*).

Plug the data from above table into your software package and try to reproduce the results below (for didactical reasons, do not include “previous_confounder” as this leads to perfect predictions in this test example); i.e. regress “treatment” (dependent variable) on “confounder” and “static_confounder” (independent variable).

*Cumulative Inverse*

*Result from Probability probability probability*

*prediction of of of*

*(Estimated “observed” “observed” treatment*

*... ... Probability) treatment treatment weight*

subject_

id time treatment propensity iptw

➀

➁

➃

*... * ** ** ***

2 ... 1 1 ... 0.34 0.34 0.34 3.0

➂

2 ... 2 1 ... 0.34 0.34 0.114 8.8

2 ... 3 1 ... 0.34 0.34 0.038 26.1

**2** ... **4 1** ... **0.34 0.34 0.013 77.5**

2 ... 5 0 ... 0.34 1-0.34 0.009 116.9

2 ... 6 0 ... 0.34 1-0.34 0.006 176.4

2 ... 7 0 ... 0.34 1-0.34 0.004 266.0

**2** ... **8 0** ... **0.34 1-0.34 0.002 401.3**

2 ... 9 0 ... 0.65 1-0.65 0.001 1153.1

**2** ... **10 0** ... **0.65 1-0.65 0.000 3313.6**

3 ... 1 0 ... 0.26 1-0.26 0.74 1.4

3 ... 2 0 ... 0.26 1-0.26 0.546 1.8

3 ... 3 0 ... 0.26 1-0.26 0.404 2.5

3 ... 4 0 ... 0.26 1-0.26 0.299 3.3

**3** ... **5 0** ... **0.26 1-0.26 0.221 4.5**

3 ... 6 1 ... 0.57 0.57 0.125 8.0

3 ... 7 1 ... 0.57 0.57 0.071 14.2

**3** ... **8 1** ... **0.57 0.57 0.040 25.1**

*...*

*Descriptions of variables introduced in the previous tables are not shown again.*

*Arrows show processing steps.*

** Result from estimation*

*** Values to be successively (as shown by the arrows) calculated from the estimation output*

Note that larger weights indicate a lower probability of treatment given a specific time-varying treatment and confounder history. In other words: If most individuals in the population with exactly this history were treated, another individual, however, was not treated, this individual would then receive a larger weight. Some weights in patient ID #2 are untenably large (see heading “Caveat: Unstable inverse probability weight” below).

**II.) “Outcome model”**

One performs a regression of outcome (dependent variable) on treatment (independent variable) – as one would have in a “conventional” analysis (typically a Cox-regression; see the heading “Model specification: Modeling survival” below for some important constraints).

However, in this regression analysis one does not include (i.e. adjust for) the confounder variables, both measured (e.g. “confounder”) and lagged (e.g. “previous_confounder”), nor any treatment lags.

In this prototypical form, marginal structural models would include only a term for the outcome (i.e. “Disability”, a term for the status of time-varying treatment (i.e. “Physical activity”) and a term for elapsed time (i.e. “Quarter of a year running count”). And: the IPTW as a *sampling weight.*

Because weights induce a within-subject correlation, one must use either robust variance estimation or bootstrap methods, taking clustering into account to calculate “correct” standard errors.

* Practical implementation

Stata:
 logistic outcome treatment time [pw=iptw], vce(cluster subject_id)

SAS:
 proc genmod {…} model outcome=treatment time / dist = binomial;
 weight=iptw class=subject_id {…};
 repeated subject=subject_id / corr=ind

R (when not using LTMLE package):
 There are different ways to achieve the goal depending on the estimation command for binary outcomes used. The command must allow sampling weights and accommodate for within-subject correlation.

**III.) The final MSM**

The weighted outcome model is then the “marginal structural model”!

Ignoring the time variable for didactical reasons (i.e. “Quarter of a year running count”; with only two subjects, estimation would show perfect determination if on includes this variable), the “treatment” with “physical activity” would reduce the odds of disability to 0.12 (95%CI 0.00 – 3.27, p=0.21) – using the example data.

## Characteristics of the data: Model specification

Whereas above “recipe” is rather trivial, its operationalization in a realistic setting is more cumbersome.

**• Caveat: Unstable inverse probability weight**

The predicted weights are often “unstable” (i.e. very large; as demonstrated in the example above). In a perfect scenario the IPTWs (dividing the predicted denominator by 1) would have a mean close to 1 and a narrow variance (see Robins et al. for a more technical explanation of why weights unfortunately often do not behave in such a way [8]). This is a prerequisite for an adequate estimation of MSMs. It, in fact, reflects correct model specification in a large sample. The example above, therefore, will not reflect any valid treatment effect estimation.

**a.) Stabilization**

Unfortunately, the native IPTW rarely performs “well”. A remedy is to “stabilize” the weights.

Practically, this amounts to replacing the IPTW “numerator” (i.e. the “1”, see heading „Process of estimation – I.iv.” above) by an estimation that is exactly the same as the estimation of the IPTW “denominator”, with an important omission: The numerator is derived by regressing treatment history *only* (and not the treatment *and* confounder histories as before) on the observed treatment at a given time-point – using the steps of the „Process of estimation – I.i. through to I.iii”. The new numerator will then be some value smaller than 1.

The stabilized IPTW is formed by dividing the (new) numerator by the (previous) denominator row-wise – effectively reducing the value of the IPTW.

See Cole and Hernan for an explanation of why this works and furthermore an easily readable best-practice tutorial of how to construct IPTWs [9].

Note the subheading “Caveat: Treatment model variables – A necessary reformulation of the treatment […]” on page 18 for a necessary reformulation of the IPTW stabilization given certain prerequisites.

**b.) Truncation**

If a mean of close to 1 is achieved, but some stabilized weights are left with large outliers, truncation [9] or normalization [10,11] may be necessary, in a trade-off between bias and precision.

**• Caveat: Treatment model variables**

The stability of the inverse probability of treatment weight is fundamentally dependent on the variables that are included in its estimation.

In fact, if *all* confounding variables that determine treatment “allocation” were known, were measurable and had been measured without error (latter pre-conditions would lead to a situation where there is “no unmeasured confounding”; treatment allocation would then be “conditionally exchangeable” given the measured confounders – see Hernán [12] for a very short explanation of what is meant by “exchangeability”) and one knew *exactly* how these variables were to specified (e.g. are there interactions between confounders, what is their correct functional form, etc.) in the estimation model, it would be possible to estimate “perfect” IPTWs and construct an MSM that then estimates unconditional population causal effects – analogous to estimates from randomized trials.

Unfortunately, one does not have all the information. Rather one is often agnostic as to which variables are salient confounders and which variables – such as intermediates – may in fact introduce further bias if included in the modeling of the IPTW [13].

(Note that “exchangeability” is one of three prerequisites to validly identify causal effects (and to be able to interpret the results from an MSM as being causal), the second being a consistent definition of the causal contrast (see the “Discussion” section of the Main manuscript) [14]. The third condition – positivity – implies that there is measured data available for every level of treatment and every level of treatment at every level of every confounder (more correctly: that there is a non-zero *probability* of having received each of the latter combinations) [15]. An example for a situation in which the positivity assumption may have gone on to be violated was introduced above: Had one used months instead of coarsening to quarters of one year, certain combinations of treatment and confounding would not have materialized (and in fact, may not have been possible).)

As such one must go through a process of model selection. As mentioned in the Main manuscript, “A confounder is considered to be a variable that is a common cause for both treatment and outcome.”. How does one identify a confounder? Is it a purely statistical condition (which is difficult if not impossible to determine in a time-varying scenario)? Again referencing Hernán et al. [16]: Expert knowledge may need to influence the decision if or if not a variable is indeed defined as a confounder.

In our case (Table 1 in the Main manuscript) we – in a first step – included those variables that were theoretically deemed to be potential confounders. And then – again as a trade-off between bias and precision (estimation may not be feasible given certain model complexities) – went on to include confounders *and* those variables that were associated with *outcome only* in univariate analysis, given a significance of p<0.1 (a completely arbitrary cut-off). Note, that the latter contradicts the proposition that the IPTW is a *treatment* weight.

***a.) Selecting variables associated with treatment and/or outcome***

However, it has been shown in simulation studies that models that include variables that strongly predict treatment (and are not confounders) in fact are prone to increased bias [17]. Moreover, including variables associated with outcome only (i.e. pure risk factors for outcome), may be beneficial when estimating the treatment model.

***b.) Time-invariant variables***

The instructions for estimation step “I.i.” (under the heading „Process of estimation”) for the treatment model noted that one prototypically includes treatment and confounder history only. However, in practice the IPTW estimation should also include time-invariant variables (e.g. static variables such as “eye color”) and the baseline measurements of confounders (i.e. pre-treatment).

There are reasons for doing so. Firstly, by ignoring salient confounders one runs the risk being left with substantial residual confounding – and therefore risking a violation of the exchangeability prerequisite.

Treatment/confounder interactions and “dynamic treatment regimens”

Secondly, there may be a more hypothesis-driven necessity. Consider following query: “What is the effect of initiating “physical activity” sometime after baseline if and only if the body mass index surpasses a certain threshold?”. This type of question can be subsumed under the heading “dynamic treatment regimens” and involves an interaction between treatment and confounder histories. Unfortunately, traditional MSMs cannot handle this type of question (Daniel et al. discuss alternative strategies for these kinds of dynamic situations [2]) – yet they are well suited to answer *static* treatment/confounder interactions such as: “Does the degree of white matter pathology at baseline lead to different risks of transition to disability given treatment with physical activity?”, i.e. the effect-modification between the time-varying treatment variable and a static variable.

A necessary reformulation of the treatment (heading „Process of estimation: steps I.i. – I.iv.”) and outcome models (heading „Process of estimation: step II.”) when including non-time-varying variables in the estimation of the IPTW

If static variables are to be added to the estimation of the IPTW, they are included both in the estimation of the denominator *and* the numerator. Consider first the basic unstabilized weight (see heading „Process of estimation – step I.i.”): One would include as further independent variables the data for e.g. pre-treatment measurements of confounders (as illustrated in the tables above). Doing so imperatively necessitates that when stabilizing the IPTW (heading “Caveat: Unstable inverse probability weight” above), that one includes these same variables in the estimation of the numerator [9]. Remember that MSMs theoretically allow an estimation of *unconditional* population causal effects. Unfortunately, having these variables in the numerator of the IPTW breaks unconditionality and re-introduces confounding. This must be dealt with by then also adding these variables to the *outcome model* (which then may prevent a fully causal interpretation of the resulting MSM (see Kaufmann [18] for a clarification)). When modeling *static* treatment/confounder interactions, the outcome model includes that interaction as a further term.

* Practical implementation

Stata:
 logistic outcome treatment time *static_confounders* [pw=iptw], vce(cluster subject_id)

SAS:
 proc genmod {…} model outcome=treatment time *static_confounders* / dist = binomial;
 weight=iptw class=subject_id {…};
 repeated subject=subject_id / corr=ind

R (when not using LTMLE package):
 Include *static_confounders* in the estimation command chosen.

**• Caveat: Missing data**

Missing data in large longitudinal studies is not only common, it is ubiquitary. Dealing with missing data in the context of MSM requires equal trade-offs as would be the case when analyzing these datasets using other estimation approaches. Dedicated MSM missing data literature is sparse with some notable exceptions [19,20]. Interesting is the fact that one could consider missingness of confounders as a “censoring” mechanism (see heading “Characteristics of the data: Censoring and other weights” below) and construct inverse probability weights of the propensity of a variable being missing to adjust appropriately. Other, perhaps more versatile approaches include multiple imputation [19] – though model specification (e.g. should one include lagged data, to what degree back in time to, impute a missing confounder, etc.) remains somewhat untenable. When treatment data is missing, simulation studies point out that a complete-case analysis may lead to the least degree of bias [20].

## Model specification: Modeling outcome other than survival; non-dichotomous treatment

**• Outcomes other than survival**

Although the most common implementation of MSMs to date has been to investigate questions related to survival (“marginal structural survival models”), modeling can be easily extended to scenarios other than those related to time to failure associated effects [21], such as dichotomous (e.g. “Do subjects reporting treatment with “physical activity” perform better after three years?”; see Bodnar et al. [22]) or continuous outcomes (e.g. “On average, was walking speed in meters per second faster in subjects reporting treatment with “physical activity” after three years?”). Moreover, it is possible to investigate repeated-measures outcomes [23]: “How much does the walking speed change per year of follow-up in subjects reporting treatment”. The outcome model must then be adapted accordingly (e.g. logistic regression, repeated-measures analysis, etc.).

**• Non-dichotomous treatment**

All these effects can represent causal contrasts of the type “What would have happened if the same subject had been treated with an alternative treatment?”. However, although treatments do not have to be dichotomous, individual comparisons nonetheless must be interpretable as causal contrasts: Multiple treatment categories – either ordered (e.g. “mild”, “moderate” or “intense” treatment with “physical activity”; there are then three causal contrasts that one has to look at separately: “mild” vs. “moderate, “mild” vs. “intense” and “moderate” vs. “intense”) or unordered (e.g. treatment with “walking”, “sit-ups” or “cycling”) – can be used (see Nandi et al. for an example [24]), as can continuous treatments (see Zhang et al. for a practical illustration [25]). In the multiple category case, weights would be predicted after e.g. ordered or multinomial logistic regression in the same way as in the dichotomous case explained above, as the probability of having received one’s actual treatment; the continuous case requires knowledge of the treatment’s probability density function – see Naimi et al. for an explanation [26].

Note that non-survival outcome and non-dichotomous treatment analysis is still rare in the published literature.

## Model specification: Modeling survival

Marginal structural model analysis of the type introduced in this paper and commonly seen in the literature asks a “survival question”. As suggested, one would expect to use e.g. proportional hazard methodology including the specific weight estimated for each subject’s specific follow-up time (i.e. the weight is not stable across time-points within a subject but rather varies as demonstrated in the table above) to estimate survival under treatment.

We used Stata 12.0 as our statistical package.

Pooled logistic regression as a work-around

Unfortunately, neither Stata’s nor SAS’s conventional survival analysis commands allow weights that vary within a subject (R seems to be able to handle this scenario [6]). This is the reason why one will find most papers analyzing marginal structural model for survival analysis employing a method called “pooled logistic regression”. Pooled logistic regression is a “normal” logistic regression set-up with data per patient organized in “discrete time intervals”. This reflects the somewhat awkward data set-up with replications introduced above.

It can be shown that if the proportion of failures is small per discrete time interval (i.e. <10%) [27], estimates from a “pooled logistic regression” approximate those from a Cox proportional hazard approach (the odds ratios are interpretable as hazard ratios).

Note if software did indeed allow for individually varying weights for Cox-models, one could incorporate exact survival times on a continuous scale, rather than coarsening outcome times to aggregates as shown above by defining each interval as a quarter of a year (Recent attempts have demonstrated that one can in fact use weighted Cox proportional hazard methods with standard software if certain constraints are applied [10].).

## Characteristics of the data: Censoring and other weights

Commonly individuals in the longitudinal setting are lost to follow-up or experience a non-study outcome before the end of observation. To avoid introducing selection bias given that some constellation of treatment and confounding is preferentially associated with this scenario, (informative) censoring must be taken into account.

As noted above, weights can be used to account for missingness. More commonly they are used to account for censoring.

To do so one constructs an “inverse probability of censoring weight” (IPCW) in the same mechanistic way as the IPTWs were estimated (including a stabilization if necessary) – with one important difference. The dependent variable in estimation step “I.i.” (under heading „Process of estimation”) was treatment. Now one regresses an indicator of the status of censoring (dependent variable) on treatment status, a lag of the treatment variable and present and past states of confounding variables (independent variables). The censoring variable will typically be 0 in the case of remaining uncensored and 1 if the next event is censoring. Censoring weights therefore reflect the present probability of remaining uncensored up to the next period in time, given treatment and confounder history (therefore IPCWs are more precisely “inverse probability of remaining under follow-up weights”).

The IPTWs and IPCWs (or any other weights that may have been introduced) are combined for a final weight (this then is the “inverse probability weight” (IPW)) by simply multiplying them together at each observation (i.e. row-wise) – this final weight is then used in the outcome model as above.

## Marginal structural models and statistics: Detailed methods

In the Main manuscript we constructed MSMs to investigate the effect of self-reported “physical activity” (the treatment) on the transition to disability (the outcome) in a three year longitudinal study, also investigating the interaction between treatment and baseline white matter pathology. In the following, we highlight the implementation of the analysis corresponding to above manual’s headings and provide further details.

**• Headings: “Data preparation” and “Caveat: Missing data”**

Data was replicated as explained above, as we opted to estimate a marginal structural model for survival analysis using a pooled logistic regression approach.

Data was available at baseline and on at most three occasions thereafter. We chose to use intervals of one quarter of a year. Subjects were followed up for a maximum of 42 months (14 quarters; some individuals were seen after the planned three year period), any later observations were ignored because outcomes and confounder variability became sparse (violating positivity).

We included as further variables, lags of time-varying variables (treatment and confounders) with data from the immediately previous follow-up.

Table 1 in the Main manuscript highlights missing values of most time-varying variables (approximately between 20 and 25% of possible data point were missing – i.e. in the time period in which individuals were under observation, either up to transition to disability, or lost to follow-up or death, or up the end of the observation period in those individuals remaining in the study). For sake of simplicity, we used the “last observation carried over” method to fill missing values. We acknowledge that this may have introduced bias.

**• Headings: “Caveat: Treatment model variables”, “Caveat: Unstable inverse probability weight” and „Process of estimation: steps I.i. – I.iv.”**

Iterative process of treatment model variable selection

The selection of variables included in the treatment model was driven by 1.) the availability of variables in the original study, 2.) an educated choice thereof of variables deemed previously to potentially confound the pathway from treatment to outcome, 3.) limitations in model stability. (These same variables were used for the estimation of the censoring weight (IPCWs). Note that this will not be adquate for the estimation of the IPCWs, as a set of other variables may be associated with the censoring process. Again. for reasons of simplicity, we opted not to perform this extra estimation step.) The final IPW was calculated by multiplying the IPTW with the IPCW data row-wise.

In a first step we included all variables selected under 2.), irrespective of their significance of association with treatment or outcome in the univariate analysis (see all variables included in the Table 1 in the Main manuscript). Estimation of the IPW including all these variables was unfeasible as the logistic regression models did not converge (a practical point that may render MSM modeling a tedious exercise). We also included theoretically plausible interactions in further sensitivity analysis; as they did not ameliorate the IPW estimation, they do not appear in the final model.

We then limited the variables to those associated with either treatment or outcome (or both), choosing a p value of <0.2. This cut-off has no inherent value other than that it was chosen arbitrarily. (Stabilized) IPWs from this model remained very large, including severe outliers. Remember that – colloquially – very large weights signal inherent problems with the IPW model, including violations of positivity [9].

As introduced above – though contradictory to the notion that one is estimating weights for treatment – outcome variables may be more adequate when generating IPWs; we therefore included variables that were associated with both treatment and outcome (i.e. potentially true confounders) or associated with outcome only; latter variables are also subsumed under the term “potential confounders” for sake of simplicity.

When removing further variables by raising the p threshold to p<0.1, an “adequate” average IPW was achieved, necessitating a rather narrow truncation of outlier weights at the 1st and 99th percentile.

Final treatment model

Latter prerequisites led to he final treatment model which included those 13 variables explicitly marked in the last column of Table 1 in the Main manuscript.

These were: “age at baseline” (time-invariant); “level of education” (time-invariant); “marital status” (time-varying); degree of baseline “white matter pathology” (time-invariant); episode of “stroke” after ascertainment, irrespective of severity (time-varying); “atrial fibrillation” (time-varying); “angina pectoris” (time-varying); self-reported “anxiety or depressed mood” (time-varying); “complaints of gait” (time-varying); self-reported “history of falls” (time-varying); diagnosed “major depressive episode” (time-varying); cognitve status measured via the “MMSE” (time-varying); self-reported “syncopal episodes” (time-varying).

Referencing the “Methods” section in the Main manuscript:
The denominator of the IPTW “included both static (e.g. “level of education” or “white matter pathology” at baseline) and time-varying variables (e.g. “history of falls”). We also included baseline values and lags (i.e. data of the previous follow-up time-point) of time-varying variables (potential confounders *and* the treatment variable). Moreover, the time variable as quarters of a year from baseline and a spline function of the time variable were entered as linear terms.”
“The numerator […] included static variables, the baseline variables for time-varying variables and the treatment history only (i.e. omitting follow-up data for the potentially confounding variables and their lags); and the time variable and its spline.”

Estimation

For denominator and the numerator variable set separately: “[…] treatment “physical activity” was regressed on these variables using logistic regression, followed by the prediction of “observed” treatment. The individual-specific “cumulative propensity” was calculated thereafter for each observed time-point. […] The (row-wise) quotient is then the so-called “stabilized” (inverse) treatment weight.“

Note that all covariates were entered the covariates of the treatment models as linear predictors. Inclusion of a spline term for the time variable acknowledges the assumption that the intercept is a smooth function, rather than separate for each time point [28].

Final IPW characteristics

The final average of stabilized IPWs for our model of choice was 1.8 (95% CI 1.28 – 2.37; median 0.93). Weights were truncate at the 1th and 99th percentiles to reduce variability given outliers (maximum stabilized weight before truncation was 1389.4 (however, only three estimated weights were larger than 100)). The final truncated stabilized IPWs had an average of 1.25 (95% CI 1.21 – 1.29) and had a ceiling of 11.51.

**• Heading: „Process of estimation: step II”**

The outcome model included the current value of treatment (plus an interaction term with the variable for the baseline degree of white matter pathology), the baseline variables of treatment and counfounders, and all static variables as independent variables plus an indicator for time passed since study start and its spline. The IPW was included as a sampling weight.

# Figure

## Time-varying treatment, time-varying confounding and the inverse probability of treatment weight


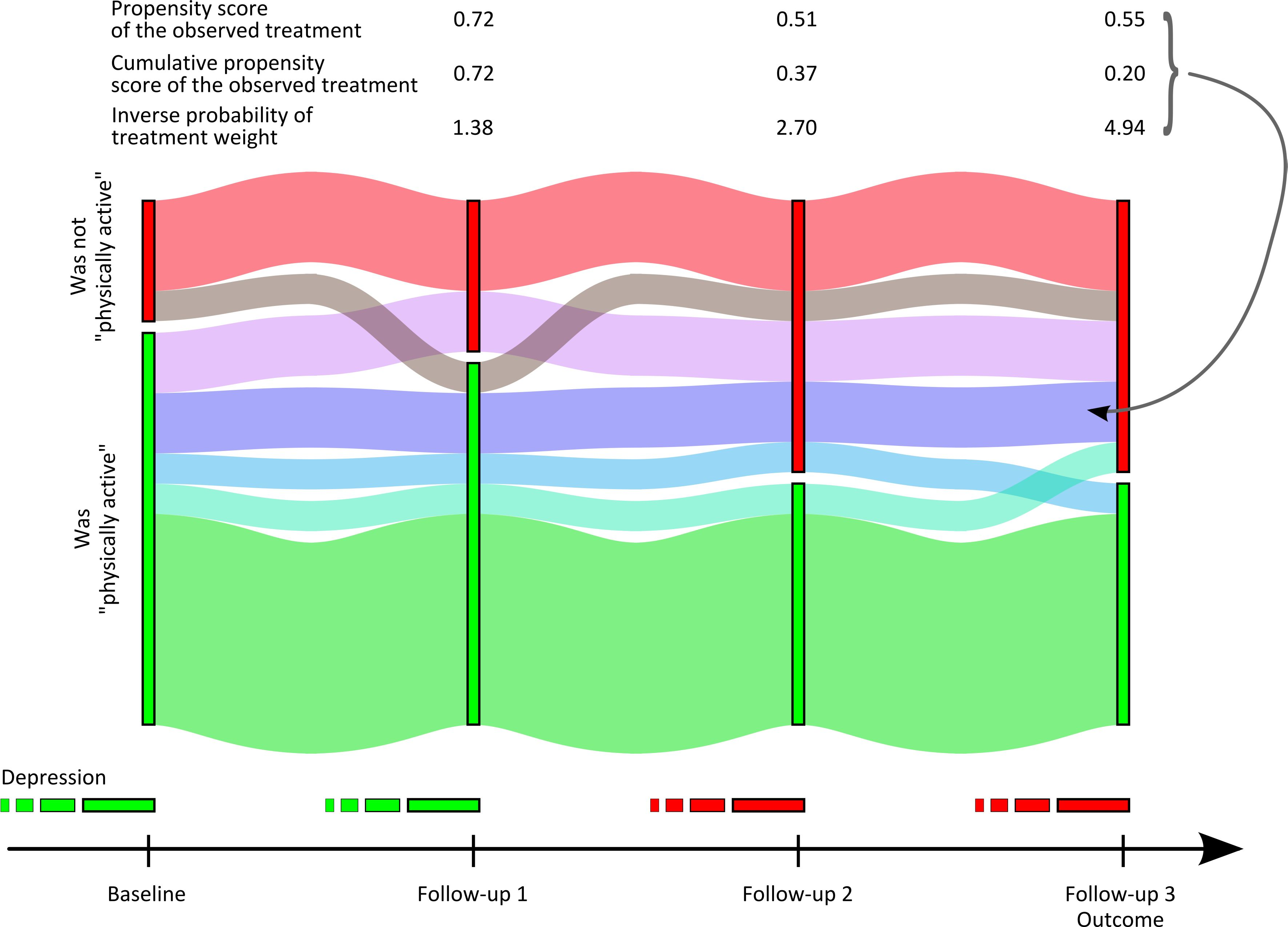


Subjects will have different patterns of time-varying confounding, unless their confounder status is exactly identical (indicated by segments of the same color).

For didactical purposes this Figure summarizes the status of treatment with “physical activity” in 17 subjects that were “depressed” at baseline and the first follow-up, but did note depressive symptoms at follow-ups 2 and 3.

E.g. seven of these subjects remained “physically active” throughout the follow-up period (wide green segment, bottom row of the alluvial plot), 3 subjects remained physically inactive (narrower red segment, top row). Some subjects varied in their respective “treatment”. For example, 2 subjects started out with “physical activity” at baseline and follow-up 1, then report inactivity at the second and third follow-up (purplish segment in the middle).

Note that most subjects (13 of 17) started out physically active at baseline (vertical bright green box to the left); most subjects turned physically inactive on follow-up 3 (9 of 17). If in fact the time-varying confounder status “depression” is associated with the time-varying status of subsequent “treatment” with “physical activity”, this deterioration seems plausible.

The Figure also illustrates the inverse probability of treatment weighting mechanism. The propensity score for each individual – correctly, the propensity score for each group of individuals with identical covariate and treatment histories (demonstrated in the Figure for those 2 subjects highlighted by the purplish segment in the middle) – summarizes the probability of receiving ones own treatment given previous treatment and covariate history.

In essence, for an individual in the “purplish” group, the propensity of having received the observed treatment “physically active” at follow-up 1 is the predicted probability (of being treated) given a logistic regression with treatment as the dependent variable, previous treatment (in this case the status at baseline, i.e. “physically active”) and the static value of baseline treatment, the previous value of the confounder “depression” (at baseline, i.e. “not depressed”) and the static value of the confounder at baseline as the independent variables. See the headings under „Process of estimation” for a software command prototype.

In this case it equals 0.72. At follow-up 2 the subject stops being physically active. As such the propensity of having received the “observed” treatment “not physically active” is the predicted probability subtracted from 1 (i.e. 0.51). The inverse probability of treatment weight (IPTW) is calculated from the cumulative propensity score of observed treatment (i.e. 0.72 at follow-up 1, 0.72×0.51=0.37 at follow-up 2 and so on) by dividing it by 1 (i.e. 1/0.72=1.38, 1/0.37=2.70 and so on).

# References

1. Fewell Z, Hernán MA, Wolfe F, Tilling K, Choi H, Sterne JA. Controlling for time-dependent confounding using marginal structural models. Stata J. 2004;4: 402–420.

2. Daniel RM, Cousens SN, De Stavola BL, Kenward MG, Sterne JAC. Methods for dealing with time-dependent confounding. Stat Med. 2013;32: 1584–1618.

3. Roger Logan, Eric Tchetgen, Miguel A. Hernan. %MSM: SAS software for survival analysis using marginal structural models [Internet]. 2004. Available under: http://cdn1.sph.harvard.edu/wp-content/uploads/sites/148/2014/07/msm.zip

4. Crowson CS, Schenck LA, Green AB, Atkinson EJ, Therneau TM. The Basics of Propensity Scoring and Marginal Structural Models [Internet]. 2013 Aug. Report No.: Technical Report Series No. 84. Available under: http://www.mayo.edu/research/documents/biostat-84-pdf/DOC-20024406

5. Faries DE, Kadziola ZA. Analysis of longitudinal observational data using marginal structural models. Analysis of Observational Health Care Data Using SAS Cary, NC: SAS Institute Inc. 2010. pp. 211–230.

6. van der Wal WM, Geskus RB. Ipw: an R package for inverse probability weighting. J Stat Softw. 2011;43 [Internet]. Available under: http://www.jstatsoft.org/v43/i13/paper

7. Schwab J, Lendle S, Petersen M, Laan M van der, Gruber S. ltmle: Longitudinal Targeted Maximum Likelihood Estimation [Internet]. 2014. Available under: http://cran.r-project.org/web/packages/ltmle/index.html

8. Robins JM, Hernán MA, Brumback B. Marginal structural models and causal inference in epidemiology. Epidemiology. 2000;11: 550–560.

9. Cole SR, Hernán MA. Constructing inverse probability weights for marginal structural models. Am J Epidemiol. 2008;168: 656–664.

10. Xiao Y, Abrahamowicz M, Moodie EEM. Accuracy of conventional and marginal structural Cox model estimators: a simulation study. Int J Biostat. 2010;6: Article 13.

11. Karim ME, Gustafson P, Petkau J, Zhao Y, Shirani A, Kingwell E, et al. Marginal Structural Cox Models for Estimating the Association Between β-Interferon Exposure and Disease Progression in a Multiple Sclerosis Cohort. Am J Epidemiol. 2014;180: 160–171.

12. Hernán MA. Beyond exchangeability: the other conditions for causal inference in medical research. Stat Methods Med Res. 2012;21: 3–5.

13. Schisterman EF, Cole SR, Platt RW. Overadjustment bias and unnecessary adjustment in epidemiologic studies. Epidemiol Camb Mass. 2009;20: 488–495.

14. Cole SR, Frangakis CE. The consistency statement in causal inference: a definition or an assumption? Epidemiology. 2009;20: 3–5.

15. Westreich D, Cole SR. Invited commentary: positivity in practice. Am J Epidemiol. 2010;171: 674-677-681.

16. Hernán MA, Hernández-Díaz S, Werler MM, Mitchell AA. Causal knowledge as a prerequisite for confounding evaluation: an application to birth defects epidemiology. Am J Epidemiol. 2002;155: 176–184.

17. Lefebvre G, Delaney JAC, Platt RW. Impact of mis-specification of the treatment model on estimates from a marginal structural model. Stat Med. 2008;27: 3629–3642.

18. Kaufman JS. Marginalia: comparing adjusted effect measures. Epidemiology. 2010;21: 490–493.

19. Moodie EEM, Delaney JAC, Lefebvre G, Platt RW. Missing confounding data in marginal structural models: a comparison of inverse probability weighting and multiple imputation. Int J Biostat. 2008;4: Article 13.

20. Shortreed SM, Forbes AB. Missing data in the exposure of interest and marginal structural models: a simulation study based on the Framingham Heart Study. Stat Med. 2010;29: 431–443.

21. Robins JM. Marginal structural models versus structural nested models as tools for causal inference. Statistical models in epidemiology, the environment, and clinical trials. Springer; 2000. pp. 95–133.

22. Bodnar LM, Davidian M, Siega-Riz AM, Tsiatis AA. Marginal structural models for analyzing causal effects of time-dependent treatments: an application in perinatal epidemiology. Am J Epidemiol. 2004;159: 926–934.

23. Hernán MA, Brumback BA, Robins JM. Estimating the causal effect of zidovudine on CD4 count with a marginal structural model for repeated measures. Stat Med. 2002;21: 1689–1709.

24. Nandi A, Glymour MM, Kawachi I, VanderWeele TJ. Using marginal structural models to estimate the direct effect of adverse childhood social conditions on onset of heart disease, diabetes, and stroke. Epidemiology. 2012;23: 223–232.

25. Zhang Y, Thamer M, Cotter D, Kaufman J, Hernán MA. Estimated effect of epoetin dosage on survival among elderly hemodialysis patients in the United States. Clin J Am Soc Nephrol CJASN. 2009;4: 638–644.

26. Naimi AI, Moodie EEM, Auger N, Kaufman JS. Constructing inverse probability weights for continuous exposures: a comparison of methods. Epidemiology. 2014;25: 292–299.

27. D’Agostino RB, Lee ML, Belanger AJ, Cupples LA, Anderson K, Kannel WB. Relation of pooled logistic regression to time dependent Cox regression analysis: the Framingham Heart Study. Stat Med. 1990;9: 1501–1515.

28. Vittinghoff E, Glidden DV, Shiboski SC, McCulloch CE. Regression Methods in Biostatistics: Linear, Logistic, Survival, and Repeated Measures Models. Springer Science & Business Media; 2012.
